# Cardiac amyloidosis: The great masquerader

**DOI:** 10.21542/gcsp.2018.18

**Published:** 2018-06-30

**Authors:** Jubran Rind, Nagib Chalfoun, Richard McNamara

**Affiliations:** 1 Michigan State University School of Human Medicine; 2 Spectrum Health Frederik Meijer Heart & Vascular Institute

## Abstract

Cardiac amyloidosis is an elusive condition that is notorious for mimicking various cardiovascular conditions that present with left ventricular hypertrophy (LVH). The hypertrophy in amyloidosis is typically diffuse; however, rare reports of echocardiographic resemblances with hypertrophic cardiomyopathy (HCM) exist, such as asymmetric septal hypertrophy and left ventricular outflow tract obstruction. Cardiac MRI can help differentiate amyloidosis from hypertrophic cardiomyopathy in unclear situations. This differentiation from HCM and other forms of cardiomyopathy has important treatment implications. Here we present the case of a 76-year-old man with cardiomyopathy who had echocardiographic features of asymmetric hypertrophic cardiomyopathy but was correctly diagnosed with amyloidosis with the help of cardiac MRI and ECG.

## Case presentation

**Figure 1.**
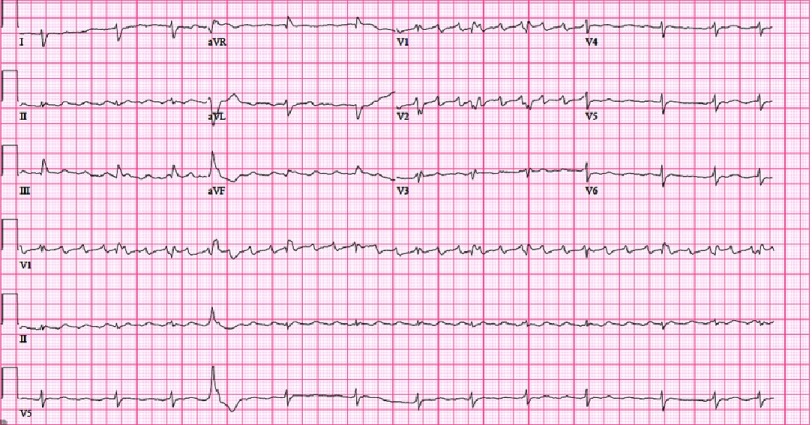
ECG showing typical atrial flutter and diffuse low voltage.

A 76-year-old man with a past medical history of coronary artery disease, hypertension, and severe septal hypertrophy suspicious for hypertrophic cardiomyopathy, presented to the hospital with progressive symptoms of heart failure. He had been suffering from worsening dyspnea and lower extremity edema for several months. Lab work over the preceding months had shown deranged liver function tests, concerning for right heart failure. He was admitted for intravenous diuretic and inotrope therapy. At his cardiology clinic appointment prior to this hospital admission, an ECG showed typical atrial flutter and diffuse low voltage [Figure 1]. A right heart catheterization showed moderately elevated right greater than left filling pressures with a right atrial pressure 18 mmHg, pulmonary capillary wedge pressure 21 mmHg and a Fick cardiac index 1.6 liters/min/m^2^. The ScvO2 was 48%.

**Figure 2.**
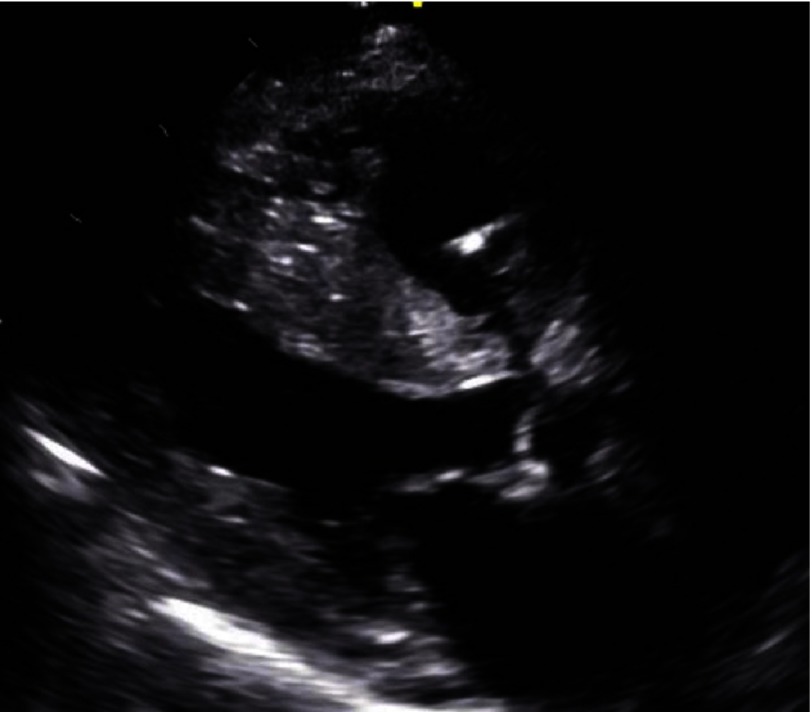
TEE at the present admission depicting left ventricular ejection fraction of 35% and septal hypertrophy.

**Figure 3.**
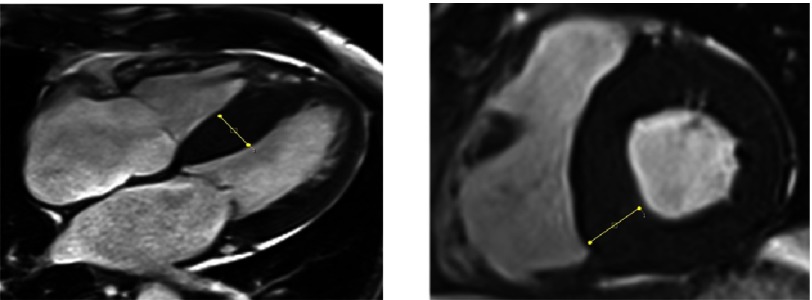
Normal left ventricular chamber size with severe, asymmetric left ventricular hypertrophy, primarily involving the septal wall with a maximum septal thickness of 27 mm.

**Figure 4.**
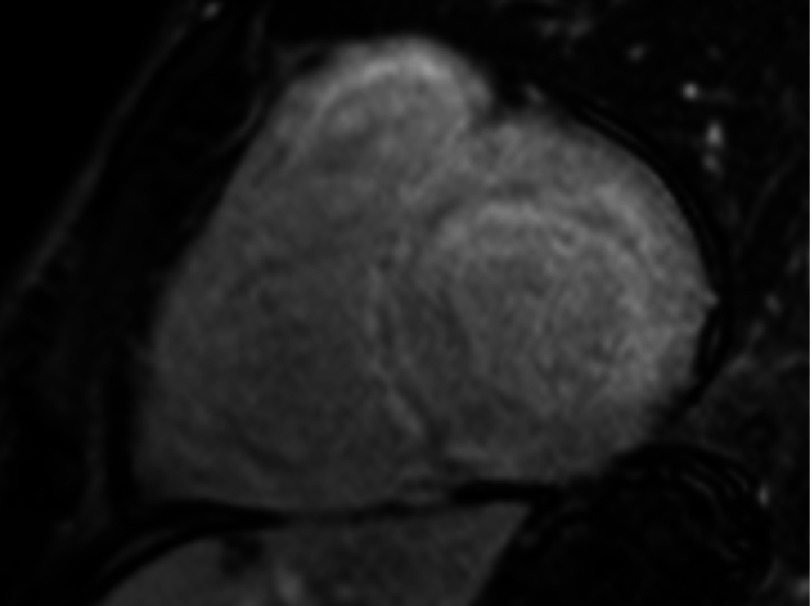
Global late gadolinium enhancement of the left ventricular myocardium, consistent with a diagnosis of cardiac amyloidosis.

Of note, a year prior to this admission, a transthoracic echocardiogram (TEE) had shown severe asymmetric septal hypertrophy (2.6 cm), suspicious for hypertrophic cardiomyopathy, but had not been further evaluated. A repeat TEE at the present admission showed a left ventricular ejection fraction (LVEF) of 35% as well as the aforementioned septal hypertrophy [Figure 2]. However, due to the significant low voltage on ECG, despite the significant LVH on echocardiogram and history of hypertension, a cardiac MRI was ordered to rule out infiltrative cardiomyopathy as opposed to hypertrophic cardiomyopathy.

The images showed normal left ventricular chamber size with severe, asymmetric left ventricular hypertrophy, primarily involving the septal wall with a maximum septal thickness of 27 mm [Figure 3]. The LVEF was measured at 40%. Also noted was diffuse, global late gadolinium enhancement of the left ventricular myocardium, consistent with a diagnosis of cardiac amyloidosis [Figure 4]. The only extracardiac clinical finding suggestive of systemic amyloidosis was carpal tunnel syndrome. Serum and urine electrophoresis did not detect a monoclonal protein, and serum free light chain ratio was low, which significantly lowered the suspicion for AL-amyloidosis. An abdominal fat pad biopsy was ordered which came back negative. Finally, an endomyocardial biopsy was performed which showed a pink amorphous interstitial infiltrate exhibiting apple green birefringence with Congo red stain, confirming cardiac amyloidosis. The subtype was likely wild-type ATTR or variant ATTR amyloidosis.

Incidentally, the cardiac MRI also found a left upper lobe mass which, on further workup, was characterized as adenocarcinoma. No further differentiation of the subtype of ATTR amyloidosis was pursued, as this would not have changed management, especially in view of the poor prognosis portended by the adenocarcinoma.

## Discussion

Cardiac amyloidosis is challenging to diagnose due to its very non-specific presentation. Systemic amyloidosis may be associated with myriad non-specific features, such as weight loss, early satiety, macroglossia, carpal tunnel syndrome, gastrointestinal bleeding, periorbital purpura, syncope, nephrotic syndrome etc. Thus, the patient may first present to any medical subspecialty.

Cardiac amyloidosis often presents as a restrictive cardiomyopathy with heart failure and progressive exercise intolerance. The pathophysiology of cardiac amyloidosis consists of deposition of autologous fibrillary proteins in the myocardial extracellular space. These proteins assume the *β*-pleated configuration and can form massive deposits, replacing myocardial tissue. They are also remarkably stable and are not effectively removed by the body, except probably in the case of AL-amyloidosis following chemotherapy^1^.

Cardiac amyloidosis can mimic hypertrophic cardiomyopathy by exhibiting asymmetric hypertrophy of the interventricular septum in rare instances^2–4^. Significant dynamic left ventricular outflow tract obstruction and presyncopal symptoms have been described in one case report^4^.

The typical 2D-echocardiographic features of cardiac amyloidosis include diffuse biventricular concentric hypertrophy, impairment in long-axis contraction on Doppler images, and normal or near normal systolic function^8^. The asymmetric septal hypertrophy seen on 2D-echocardiography in the present case was first noticed two years ago, and LVEF had deteriorated from 55% to 35% over this period. This was concerning for late onset HCM, which is a well-known phenomenon and may be detected after 60 years of age. The fact that the patient had significant LV systolic dysfunction was even more concerning because a decline in LVEF to <50% in HCM meets the criteria for end-stage or “burned-out” hypertrophic cardiomyopathy, which is associated with high mortality^5, 6^.

Also, HCM is typically associated with significant LVH on ECG. In this case, the ECG showed significant low voltage suggestive of an infiltrative myopathy instead of HCM. Therefore, a cardiac MRI was performed which showed diffuse transmural late gadolinium enhancement of both ventricles; a pattern highly characteristic of cardiac amyloidosis^7^. It also showed an LVEF of 40%.

The cardiac MRI findings prompted workup for light chain (AL) amyloidosis, which ought to be ruled out first because of its rapidly progressive course and poor prognosis in case of cardiac involvement. This is especially true if NT-proBNP and troponin are elevated^9^, as was the case with our patient. The workup for AL-amyloidosis, which included an abdominal fat pad biopsy (typically positive in >70% cases of AL-amyloidosis), was negative. Therefore, an endomyocardial biopsy (EMB) was pursued. We believe this was justified as there were no other specific extracardiac features of amyloidosis and the pattern of cardiac hypertrophy itself was quite atypical. EMB remains the gold standard for diagnosis^10^ and apple green birefringence under polarized light on Congo red staining is classically seen.

The management of cardiac amyloidosis differs significantly from other kinds of cardiomyopathy. This differentiation has important treatment implications. As far as medical management is concerned, loop diuretics are the mainstay of treatment and may be combined with aldosterone receptor antagonists. The role of ACEIs/ARBs is unclear as there are no clinical trials on their use in cardiac amyloidosis. *β*-blockers, which are known to confer a significant mortality benefit in other forms of cardiomyopathy, may in fact be detrimental in amyloidosis due to their negative chronotropic effect. In amyloid cardiomyopathy, the cardiac output is heart-rate dependent. Since the stroke volume is practically fixed due to the restrictive nature of the disease, a decrease in heart rate by *β*-blockers worsens heart failure^1^. Calcium channel blockers and digitalis are relatively contraindicated as they bind the amyloid fibrils in the myocardium and thus become concentrated to toxic levels^11, 12^.

Unlike other forms of cardiomyopathy in which an implantable cardioverter defibrillator (ICD) and cardiac resynchronization therapy are recommended in severe left ventricular systolic dysfunction, the limited data that is available about cardiac amyloidosis does not support device therapy for prevention of sudden cardiac death or improvement in left ventricular function^13^. The majority of sudden cardiac deaths in cardiac amyloidosis result from pulseless electrical activity due to electromechanical dissociation, rather than fatal ventricular arrhythmias.

Cardiac transplantation has a limited role in amyloid cardiomyopathy for several reasons. These include the multisystem involvement by amyloidosis (especially AL amyloidosis), advanced age at diagnosis (especially in senile ATTR amyloidosis), treatment-related complications, and rapid disease progression^1^. Cardiac amyloidosis has a generally poorer long-term post-transplant survival than other indications for cardiac transplant (54% compared to 75% at 5-years)^16^. Combined heart and liver transplantation has been successfully used in variant (familial) ATTR amyloidosis, because transthyretin protein is exclusively made in the liver^14^.

Large scale studies on the use of mechanical circulatory support devices, such as left ventricular assist devices (LVAD), in cardiac amyloidosis are lacking and they are infrequently attempted due to the biventricular nature of the cardiomyopathy. Right-sided heart failure is a common cardiac manifestation in patients with end-stage cardiac amyloidosis, which is an established risk factor for increased post-LVAD morbidity and mortality. Ideal treatments for biventricular failure include Bi-VAD or total artificial heart (TAH); however, there is a paucity of data regarding the benefits versus risks of biventricular circulatory support as a bridge to heart transplantation in cardiac amyloidosis. Also, the use of biventricular mechanical support as destination therapy is associated with 50% mortality at 1 year^17^. One case of successful total artificial heart implantation as a bridge to heart transplantation in a patient with wild-type ATTR amyloidosis has been described^15^.

Newer treatments targeting the underlying protein misfolding disorder have recently been introduced. Tafamidis, a drug that stabilizes the TTR molecule, is approved in Europe for amyloid associated polyneuropathy. ALN-TTR01 and ALN-TTR02 (Phase I trials) are lipid nanoparticles encapsulating an anti-TTR interfering RNA that have shown promise in the treatment of transthyretin amyloidosis^18^.

## LIST OF ABBREVIATIONS

LVH: Left ventricular hypertrophy
HCM: Hypertrophic cardiomyopathy
MRI: Magnetic resonance imaging
ECG: Echocardiogram
TEE: Transesophageal echocardiogram
LVEF: Left ventricular ejection fraction
EMB: Endomyocardial biopsy
ICD: Implantable cardioverter defibrillator
LVAD: Left ventricular assist device
TAH: Total artificial heart
